# Molecular correlation of response to pyrotinib in advanced NSCLC with *HER2* mutation: biomarker analysis from two phase II trials

**DOI:** 10.1186/s40164-023-00417-y

**Published:** 2023-06-09

**Authors:** Shiqi Mao, Shuo Yang, Xinyu Liu, Xingya Li, Qiming Wang, Yiping Zhang, Jianhua Chen, Yan Wang, Guanghui Gao, Fengying Wu, Tao Jiang, Jiao Zhang, Ying Yang, Xiang Lin, Xiaoyu Zhu, Caicun Zhou, Shengxiang Ren

**Affiliations:** 1 Department of Medical Oncology, Shanghai Pulmonary Hospital, Cancer Institute, Tongji University School of Medicine, Shanghai, 200433 China; 2 Second Ward of Oncology, The First Affiliated Hospital of Zhengzhou University, Zhengzhou, 450000 China; 3 Department of Internal Medicine, Affiliated Cancer Hospital of Zhengzhou University, Henan Cancer Hospital, Zhengzhou, 450008 China; 4 Department of Thoracic Oncology, Zhejiang Cancer Hospital, Hangzhou, 310000 China; 5 Department of Medical Oncology, Cancer Hospital of Central South University, Changsha, 410006 China; 6 Genecast Biotechnology Co., Ltd, Wuxi, 214104 China; 7 Jiangsu Hengrui Pharmaceuticals Co., Ltd, Shanghai, China

**Keywords:** Non-small cell lung cancer, *HER2* mutation, pyrotinib, ctDNA

## Abstract

**Background:**

Non-small cell lung cancer (NSCLC) with *HER2* mutation has entered into the era of targeted therapy. However, both anti-HER2 antibody–drug conjugates (ADCs) and tyrosine kinase inhibitors (TKIs) showed moderate objective response rate (ORR) and median progression-free survival (PFS). The aim of this study was to investigate the molecular features of responders to pyrotinib in advanced NSCLC with *HER2* mutation.

**Methods:**

Patients from our two previous phase II trials were pooled analyzed. Their circulating tumor DNA (ctDNA) were detected by next-generation sequencing (NGS) panels, and the correlation with the efficacy of pyrotinib was investigated.

**Results:**

This pooled analysis included 75 patients, and 50 of them with baseline plasma samples were finally enrolled with a median age of 57 years old. The overall ORR and median PFS were 28% and 7.0 months respectively. Biomarker analysis showed that 5 patients were ctDNA nonshedding. Patients with *TP53* wild type were significantly associated with higher disease control rate (97.1%vs. 68.8%, p = 0.010), PFS (median 8.4 vs. 2.8 months, p = 0.001) and overall survival (OS, median 26.7 vs. 10.4 months, p < 0.001) than those with mutations. ctDNA of nonshedding and clearance exhibited significantly longer PFS (median: 10.2 vs. 9.8 vs. 5.6 months, p = 0.036) and a trend of longer OS (median: 35.3 vs. 18.1 vs. 14.6 months, p = 0.357) than those not.

**Conclusion:**

Patients with *TP53* wild type, ctDNA nonshedding, or clearance showed superior efficacy of pyrotinib in patients with *HER2*-mutated advanced NSCLC, which might be helpful to guide the utility of pyrotinib in clinical setting.

*Trial registration:* The patients were from two registered clinical trials (ClinicalTrials.gov: NCT02535507, NCT02834936).

**Supplementary Information:**

The online version contains supplementary material available at 10.1186/s40164-023-00417-y.

## Background

Currently, NSCLC with *HER2* mutation entered into the era of targeted therapy [1, 2]. Based on the promising results of phase II trials that investigated the efficacy and side effects of ado-trastuzumab emtansine (T-DM1) [3, 4] and trastuzumab deruxtecan (T-DXd) [5], national comprehensive cancer network guideline has recommended them as options for patients with advanced *HER2*-mutant NSCLC, and the later got the approval by FDA in later line setting [6]. Meanwhile, TKIs targeting *HER2* mutation also achieved a breakthrough, pyrotinib [7–9] or poziotinib [10] showed an inspiring antitumor activity in this setting in phase II trials, and the former got the recommendation by the Chinese society of clinical oncology (CSCO) guideline. Besides, several other novel TKIs including tarloxotinib (NCT03805841) [11], TAK-788 (NCT02716116) [12] are under development.

However, both the ADCs and TKIs showed moderate efficacy, with an ORR of 31–55% and median PFS of 4.4–8.2 months [3–5, 7]. As a result, identifying the benefit population or improving the efficacy by combination is essential in the clinical setting. Currently, several strategies, such as combining with anti-angiogenesis [13, 14], immunotherapy [15], were ongoing and reported inspiring preliminary results. In this study, aiming to clarify molecular features of responders in patients of *HER2* mutant advanced NSCLC treated with pyrotinib, we collected the ctDNA and performed the biomarker analysis from the pooled analysis of our two previous phase II trials [7, 8].

## Methods

### Patients and sample collection

Patients were recruited from two phase II clinical trials of pyrotinib (ClinicalTrial.gov: NCT02535507, NCT02834936). Briefly, eligible patients with advanced *HER2*-mutant NSCLC who previously received systemic treatment were enrolled. All enrolled patients received pyrotinib 400 mg or 320 mg per day, until intolerable toxicity, disease progression, death or withdrawal of consent. Peripheral blood sample collection was performed at baseline, 40 days and 80 days after pyrotinib administration. A total of 50 patients were enrolled in the biomarker analysis. Among them, 21 pretreated tissue samples and 112 serial blood samples were collected. The study protocol was approved by ethics committees and relevant health authorities. All patients signed informed consent forms of our study.

### DNA extraction and sequencing

DNA was isolated from formalin-fixed paraffin-embedded (FFPE) tumor specimens with the TIANamp genomic DNA kit (TIANGEN, China) according to the manufacturers’ instructions. Genome DNA is extracted by TGuide S32 magnetic blood genomic DNA kit (TIANGEN, China) from peripheral blood lymphocyte (PBL), and circulating cell-free DNA (cfDNA) is extracted by MagMAX cell-free DNA isolation (ThermoFisher, USA) from the plasma sample. Fragmented DNA libraries were constructed with a KAPA HTP library preparation kit (Illumina Platform) (KAPA Biosystems, Massachusetts, USA) according to the manufacturer’s instructions. All libraries were quantified using AccuGreen high sensitivity dsDNA quantitation kit (Biotium, USA), with library size assessed on agilent bioanalyzer 2100 (Agilent, USA). DNA libraries from baseline tissue samples were captured with Panel 1, which was a designed panel spanning 769 cancer-related genes (Genecast, Wuxi, China), while DNA libraries from plasma samples were captured with panel 2, which covered exon regions of 95 genes (Genecast, Wuxi, China) related to drug resistance. The captured library was sequenced on Illumina Novaseq 6000 with paired end 150 bp mode.

### Variant calling

After filtering low quality reads by Trimmomatic(v0.36) [16], clean reads were aligned to the human reference genome (hg19, NCBI Build 37.5) with the Burrows-Wheeler aligner (version 0.7.17) [17]. Then Picard toolkit (version 2.23.0) [18] was applied for making duplicates and genome analysis tool kit (version 3.7) [19] was used for realignment. VarDict (version 1.5.1) [20] was used to call single nucleotide variant (SNV) mutations while compound heterozygous mutations were merged by FreeBayes (version 1.2.0) [21]. Sentieon software (genomics-201911) was also used to improve the detection rate of mutations in plasma samples, and the mutations were annotated through ANNOVAR [22]. Typical QC-filtering such as variant quality and strand bias was applied to the raw variant list. Additionally, variants in low complex repeat and segmental duplication regions that matched to the lowly mappable regions were defined by ENCODE [23], and variants in an internally developed and validated list of recurrent sequence-specific errors (SSEs) were removed.

### Somatic mutation filtering of tumor tissues and plasma

After filtering germline or hematopoietic origin mutations by comparing with paired normal sample, somatic mutations met the following criterions were used for the following analysis: (i) The variant allele frequency (VAF) threshold of mutations was 5% in tumor and 1% plasma; (ii) All low-frequency mutations in samples from the same patient in different time points were retained. For plasma samples, we used a pre-defined blacklist to remove false positive variants introduced for special processing of UMI data. These quality cut-offs were predetermined during the analytical validation of the internal NGS panel to optimize the test performance and measured according to sensitivity, specificity, repeatability and reproducibility.

### Statistical analysis

Chi-square test or Fisher’s exact test was used to compare the categorical variables. PFS and OS curves were estimated by Kaplan–Meier method and compared by Log-rank test. Cox proportional hazard model was performed for univariate and multivariate survival analyses to calculate the hazard ratio (HR) and 95% confidence interval (CI). Mann–Whitney U tests was introduced to analyze the significant difference of mutation frequency between defined groups. And statistical significance was defined with *P*-value < 0.05. All of the statistical analyses were performed using R V.3.6.1. and SPSS statistical software (version 22.0; IBM Corporation, Armonk, NY, USA).

## Results

### Molecular characterization of *HER2* mutated NSCLC

**Fig. 1:**
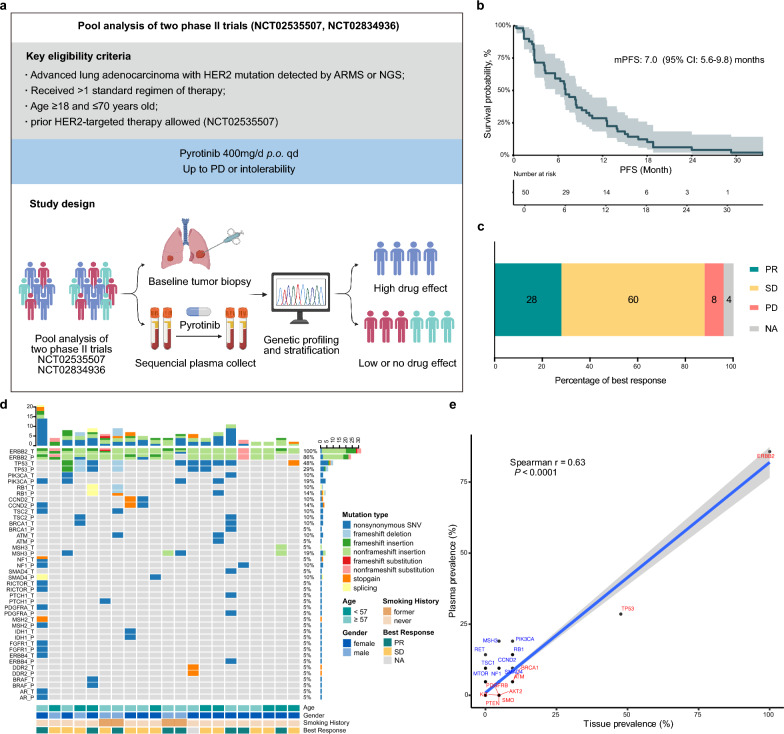
Study design and treatment efficacy of enrolled patients. **a** Key eligibility criteria and study design. **b** Kaplan–Meier estimates of PFS for enrolled patients. **c** Best response of enrolled patients. **d** Concordance analysis of high-frequency mutations in paired plasma and tumor tissue samples among 21 patients. **e** Correlation between mutation frequencies plasma samples versus tissue samples. *PFS* progression free survival, *PR* partial response, *SD* stable disease, *PD* progressive disease, *NA* not available

This prespecified biomarker analysis was performed on longitudinal samples collected from two phase II clinical trials (NCT02535507: n = 15, NCT02834936: n = 60) which evaluated the efficacy and safety of pyrotinib in *HER2*-mutant advanced lung adenocarcinoma after platinum-based chemotherapy (Fig. 1a). Baseline tumor tissue samples and serial blood samples were collected and underwent DNA sequencing. 50 patients with baseline plasma samples that were successfully sequenced were finally enrolled, including 21 pretreated tissue samples and 112 serial blood samples (pretreated: n = 50, 40 days post-treatment: n = 37, 80 days post-treatment: n = 25). Baseline characteristics were presented in Table 1. The median PFS and ORR were 7.0 months and 28%, respectively (Fig. 1b, c). The consistency of variants detection between ctDNA and corresponding tissue samples among 21 patients with paired pretreated samples was compared (Fig. 1d), with a Spearman r value of 0.63 (p < 0.0001, Fig. 1e). A positive correlation was observed between mutation count in baseline ctDNA and tumor CT volume (Spearman r = 0.43, p = 0.02, Additional file 1: Fig. S1).

**Table 1:** Demographic data and clinical characteristics of enrolled patients

Characteristic	Biomarker cohort (n = 50), No (%)	*TP53* MUT (n = 16), No (%)	*TP53* WT (n = 34), No (%)	*p* value
Median age, years (range)	57 (40–72)	53 (41–72)	61 (40–69)	0.020
Sex				
Male	21 (42.0)	6 (37.5)	15 (44.1)	0.658
Female	29 (58.0)	10 (62.5)	19 (55.9)	
Smoking histology				
Never	35 (70.0)	12 (75.0)	23 (67.6)	1.000
Former	14 (28.0)	4 (25.0)	10 (29.4)	
Current	1 (2.0)	0 (0.0)	1 (2.9)	
ECOG performance status				
0	7 (14.0)	1 (6.3)	6 (17.6)	0.406
1	43 (86.0)	15 (93.8)	28 (82.4)	
Clinical stage				
IIIB	2 (4.0)	0 (0.0)	2 (5.9)	1.000
IV	48 (96.0)	16 (100.0)	32 (94.1)	
No. of metastatic organs				
≤ 2	25 (50.0)	7 (43.8)	18 (52.9)	0.544
> 2	25 (50.0)	9 (56.3)	16 (47.1)	
Prior chemotherapy				
< 2 Lines	28 (56.0)	12 (75.0)	16 (47.1)	0.063
≥ 2 Lines	22 (44.0)	4 (25.0)	18 (52.9)	
Previous targeted therapy				
No	38 (76.0)	13 (81.3)	25 (73.5)	0.728
Yes	12 (24.0)	3 (18.8)	9 (26.5)	
Previous radiotherapy				
No	35 (70.0)	12 (75.0)	23 (67.6)	0.746
Yes	15 (30.0)	4 (25.0)	11 (32.4)	

*ECOG PS* Eastern Corporation Oncology Group

We listed the SNV and indel landscape of baseline ctDNA in Additional file 1: Fig. S2. *TP53*, *NF1*, *PIK3CA*, *RET* and *MTOR* were the top five mutant genes by frequency and were detected in 16 (32%), 10 (20%), 6 (12%), 6 (12%), 5 (10%) patients, respectively. In addition, circulating *HER2* variants were detected in 45 of 50 (90%) pretreated plasma samples. The remaining 5 patients with undetectable *HER2* variants were categorized as nonshedding tumor, with a trend of higher ORR (60.0% vs 24.4%, p = 0.126) and longer PFS (median: 10.2 vs. 6.8 months, p = 0.131), though not reaching statistical difference due to small sample size of nonshedding tumor. After comparing the clinicopathological characteristics of these two groups, we found that nonshedding tumor had a lower tumor burden (22.4 vs 53.0, p = 0.027), although the other variables were not statistically different (Additional file 2 Table S1).

### TP53 wild type associated with superior efficacy

**Fig. 2:**
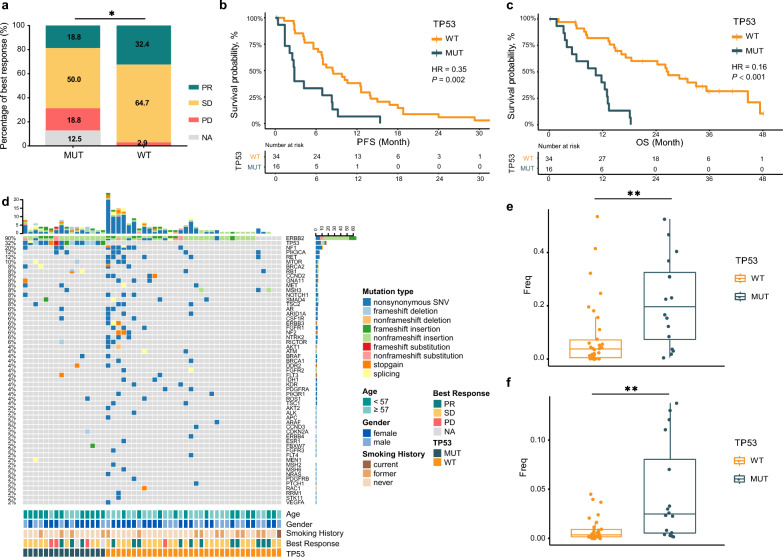
The association of *TP53* co-mutation with clinical outcomes. **a** best response of patients stratified by *TP53* co-mutation status. **b**, **c** Kaplan–Meier estimates of PFS **b** and OS **c** of patients stratified by *TP53* co-mutation status. **d** Mutation landscape in patients stratified by *TP53* co-mutation status. **e**, **f** Comparing mutation load of total gene **e** and *HER2* gene **f** in patients stratified by *TP53* co-mutation status. *PR* partial response, *SD* stable disease, *PD* progressive disease, *NA* not available, *MUT* mutant, *WT* wild type, *PFS* progression free survival, *OS* overall survival, *HR* hazard ratio, *SNV* single nucleotide variant, *Freq* frequency

As mentioned above, *TP53* was the most frequent concurrent mutation. Therefore, we further demonstrated the impact of concurrent *TP53* mutation on pyrotinib efficacy. As result, patients with wild type *TP53* were significantly associated with superior disease control rate (DCR, 97.1% vs. 68.8%, p = 0.010, Fig. 2a), PFS (median: 8.4 vs. 2.8 months, p = 0.001, HR = 0.35 95% CI 0.18–0.67, p = 0.002, Fig. 2b) and OS (median: 26.7 vs. 10.4 months, p < 0.001, HR = 0.16, 95% CI 0.07–0.35, p < 0.001, Fig. 2c) than those with *TP53* co-mutation, though the improvement of ORR (32.4% vs. 18.8%, p = 0.501) was marginal. Demographic analysis revealed that *TP53* co-mutations were more common in younger patients, with no other significant differences identified (Table 1).

**Fig. 3:**
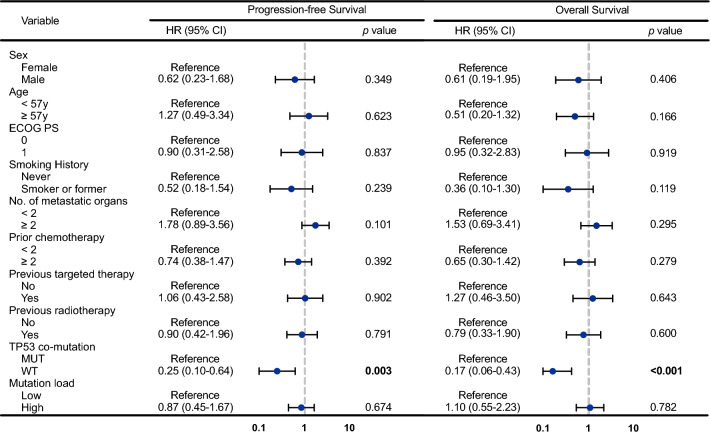
Multivariate COX regression analysis of *TP53* mutation status and clinical parameters on treatment outcomes

To characterize the discrepancy of genomic features between patients with or without *TP53* co-mutation, we performed baseline ctDNA mutation landscape in patients stratified according to *TP53* co-mutation status (Fig. 2d). Of note, patients without *TP53* mutations had lower mutation load than those with *TP53* mutations (median [P25–P75]: 3.78 [0.51–7.17] vs. 19.63 [7.32–32.51], p = 0.003, Fig. 2e). Furthermore, the mutation load of *HER2* gene was also lower in patients without *TP53* mutations (median [P25–P75]: 0.35 [0.15–0.91] vs. 2.48 [0.54–8.04], p = 0.001, Fig. 2f). To assess the predictive capability of *TP53* mutation status on treatment efficacy, a multivariable COX regression analysis that included mutation load and various clinical factors was utilized (Fig. 3). The results showed that *TP53* co-mutation was an independent risk factor for both PFS (p = 0.003) and OS (p < 0.001), while mutation load was not significantly correlated with treatment outcomes. These findings underscore the importance of considering *TP53* mutation status as a prognostic marker when evaluating treatment response.

### The association between ctDNA status and clinical outcome

**Fig. 4:**
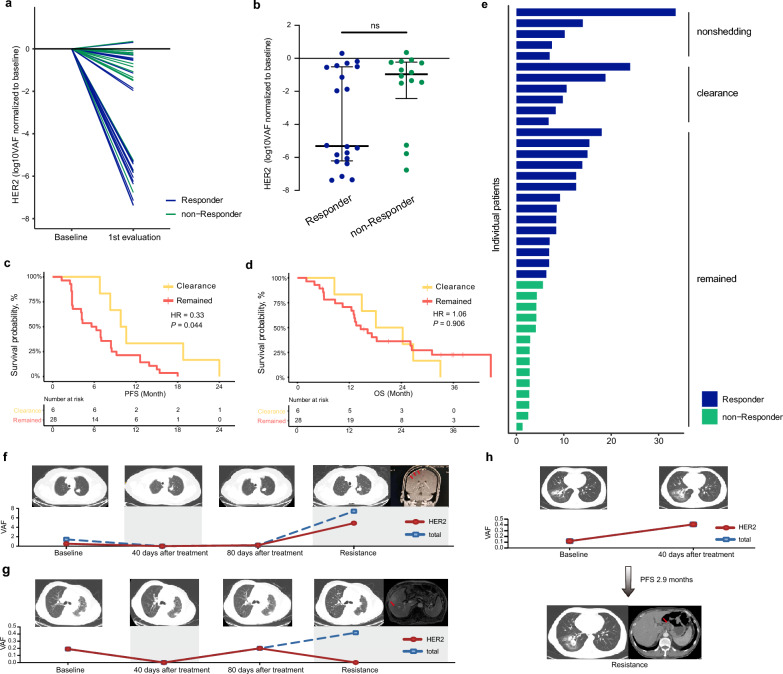
The association of ctDNA status with clinical outcomes. **a** Dynamics of *HER2*-mutant ctDNA between baseline and first radiological evaluation. **b** log10 of the change of *HER2*-mutant VAF according to response to pyrotinib treatment. **c, d** Kaplan–Meier estimates of PFS **c** and OS **d** for 34 patients stratified by ctDNA status at the first radiographic evaluation (40 days after start of targeted therapy). **e** Duration of pyrotinib treatment in patients with ctDNA nonshedding, clearance and remained. **f**–**h** Representative cases in which ctDNA can be used to monitor patients' response to pyrotinib treatment in three different conditions. *VAF* variant allele frequency, *PFS* progression free survival, *OS* overall survival, *HR* hazard ratio

Previous researches have demonstrated that dynamic change of ctDNA was associated with treatment response and survival in solid tumors treated with targeted therapy [24]. In this study, we further evaluated ctDNA clearance as a predictor of pyrotinib response. A total of 34 patients had matched baseline and first evaluation plasma samples with evaluable response data. The individual VAF changes of *HER2* mutations were presented in Fig. 4a. Patients who presented partial response (PR) or stable disease (SD) ≥ 12 weeks (defined as responder) exhibited a greater *HER2*-mutant ctDNA decrease compared with those who presented SD < 12 weeks or progressive disease (PD) (defined as non-responder), not reaching statistical significance (p = 0.056, Fig. 4b). Complete clearance (defined by conversion from ctDNA positive at baseline to ctDNA negative) of *HER2*-mutant ctDNA was observed at the first radiological evaluation in 17.6% (6/34) patients. These patients with *HER2*-mutant ctDNA clearance exhibited significantly longer PFS (median: 9.8 vs. 5.6 months, p = 0.032, HR = 0.33, 95% CI 0.12–0.97, p = 0.044, Fig. 4c) than those with *HER2*-mutant ctDNA remained but with similar OS (median: 18.1 vs. 14.6 months, p = 0.906, HR = 1.06, 95% CI 0.42–2.64, p = 0.906, Fig. 4d). Furthermore, we listed treatment duration and response to pyrotinib of patients with 3 types of ctDNA status (nonshedding ctDNA [n = 5], cleared *HER2*-mutant ctDNA [n = 6], remained *HER2*-mutant ctDNA [n = 28]) in Fig. 4e.

We presented here three cases in which ctDNA can be used to monitor patients' response to pyrotinib treatment in different conditions. Patient #12012 had decreased ctDNA VAF for both *HER2*-mutant gene and total mutant genes at both 40 and 80 days after pyrotinib treatment, and both increased at the time of PD, reflecting changes in ctDNA consistent with the efficacy evaluated by imaging (Fig. 4f). Patient #12001 had a rise in total mutant genes at the time of resistance despite no detectable *HER2* mutation, and on imaging this patient presented with stable target lesion but was evaluated as progression due to new liver metastases (Fig. 4g). Patient #05002 had significantly elevated *HER2*-mutant ctDNA at first evaluation and also showed on imaging an enlarged target lesion and new interstitial hepatogastric lymph node metastases with a PFS of 2.9 months (Fig. 4h).

## Discussion

In this study, we firstly presented the up-to-date largest cohort of dynamic ctDNA profiling in *HER2* mutant NSCLC on the basis of a prespecified biomarker analysis of two prospectively trials. We found that *TP53* wild type at baseline were independently correlated with better clinical outcomes, including superior disease control rate (p = 0.010), longer PFS (p = 0.001) and OS (p < 0.001), than those with mutation. Our study also revealed that nonshedding tumor or ctDNA clearance were associated with superior efficacy. These findings shed light on the individual targeted therapy in patients with *HER2* mutation.

Therapeutic landscape has been largely changed in patients of advanced NSCLC with *HER2* mutation [25, 26]. Doublet chemotherapy used to be the standard-of-care for *HER2*-mutated NSCLC, however, previous results including ours showed a discouraging result with the ORR of 10–43.5% and median PFS of 4.3–6 months [27]. Subsequently, it was also found that patients with *HER2* mutation were unsuitable for immunotherapy, such as anti-PD-1/PD-L1 monotherapy [15, 28, 29]. Encouragingly, the recent advance of ADCs such as T-DM1 [4] and T-DXd [5, 30], TKIs such as pyrotinib [7, 31] or poziotinib [10] showed inspiring antitumor activity in *HER2*-mutated NSCLC in phase II trials, which made *HER2* mutation a druggable target. However, their moderate efficacy indicated that there’s an urgent need to establish effective predictive biomarkers in the clinical practice.

As far as we know, this is the first study to investigate the predictive role of genomic alternations through the ctDNA detection in *HER2* mutant NSCLC, and we found that *TP53* mutation was associated with the inferior efficacy of pyrotinib. Previously, several studies revealed that concurrent mutations would deteriorate the anti-cancer effect of EGFR-TKIs in patients with *EGFR* mutations [32, 33]. Moreover, *TP53* mutations was further found to promote genetic evolution and accelerate occurrence of resistance both in patients with *ALK* fusion and *EGFR* mutation [34]. Similarly, this study for the first time reported that *TP53* mutation was of vital importance in the resistance of pyrotinib treatment in *HER2* mutant NSCLC. Taken together, these findings suggested that *TP53* mutations played an important role in the resistance of targeted therapy and needed to be considered as a stratification factor in study design in the future.

Moreover, we found that 10% patients had nonshedding ctDNA of lung tumors at baseline and after pyrotinib therapy. Importantly, patients with nonshedding tumors had a higher ORR of 60% and longer median progression free survival of 10.2 months. Recently, it was found that the presence of nonshedding tumors in the minimal residual disease (MRD) detection was associated with longer relapse-free survival (RFS) and higher possibility of cure [35]. Although the nonshedding observation might also be attributed to the insufficient detection sensitivity, the possible underlying mechanisms still needed to be further explored. Our findings showed that nonshedding might also be served as a potential biomarker for the superior prognosis and efficacy of pyrotinib treatment.

Additionally, we also investigated the predictive role of ctDNA dynamics. Previously, several reports including ours consistently demonstrated that ctDNA clearance after 2 cycle of chemotherapy [36] or immune checkpoint inhibitors (ICIs) therapy [37, 38] was associated with a better efficacy in advanced NSCLC, indicating that ctDNA dynamics was a useful marker for systemic therapy in lung cancer. In this study, we also observed that the pyrotinib treatment efficacy was superior in patients with ctDNA clearance after 40 days of treatment. Taken together, this study highlighted the importance of ctDNA detection and found that *TP53* wild type, nonshedding tumor and ctDNA clearance could be used to identify the patients benefit from pyrotinib treatment in NSCLC.

Several limitations must be mentioned in this study. First, the number of patients finally enrolled into the analysis was still small even this was a pooled analysis of two phase II trials. Thus, selection bias might be inevitable. Second, only blood samples were used for the biomarker analysis due to the insufficient tissues and difficulty of re-biopsy at the disease progression, some genomic information might be missed in the liquid-based NGS testing. Thirdly, currently pyrotinib was only recommended by CSCO NSCLC guidelines in the later line setting, therefore, these findings might not suitable for a wide generalization.

## Conclusions

In conclusion, our study highlighted the potential advantage of ctDNA analysis for precisive treatment of pyrotinib in patients with *HER2* mutation. We found that *TP53* mutations could accelerate the occurrence of drug resistance during targeted therapy. We also unveiled that ctDNA clearance or nonshedding tumor were associated with superior efficacy of pyrotinib treatment. These findings might be helpful to guide the clinical utility of anti-HER2 targeted therapy, which still requires further validating in the future.

## Supplementary Information

**Additional file 1: Figure S1 **Correlation between mutation count and tumor size evaluated by CT. **Figure S2** Mutation landscape of all enrolled patients.

**Additional file 2: Table S1 **Comparing demographic data and clinical characteristics between patients with shedding or nonshedding tumor.

## Data Availability

The datasets generated during and/or analyzed during the current study are available from the corresponding author on reasonable request.

## Abbreviations

NSCLC: Non-small cell lung cancer
ADCs: Antibody–drug conjugates
TKIs: Tyrosine kinase inhibitors
ORR: Objective response rate
PFS: Progression-free survival
ctDNA: Circulating tumor DNA
NGS: Next-generation sequencing
OS: Overall survival
T-DM1: Ado-trastuzumab emtansine
T-DXd: Trastuzumab deruxtecan
CSCO: Chinese society of clinical oncology
FFPE: Formalin-fixed paraffin-embedded
cfDNA: Cell-free DNA
PBL: Peripheral blood lymphocyte
SNV: Single nucleotide variant
SSEs: Sequence-specific errors
VAF: Variant allele frequency
HR: Hazard ratio
CI: Confidence interval
PR: Partial response
SD: Stable disease
PD: Progressive disease
MRD: Minimal residual disease
RFS: Relapse-free survival
ICIs: Immune checkpoint inhibitors
