# Supplementary material for: Molecular correlation of response to pyrotinib in advanced NSCLC with HER2 mutation: biomarker analysis from two phase II trials
Source: Exp Hematol Oncol. 2023 Jun 9;12:53. doi: 10.1186/s40164-023-00417-y (PMC10251549; doi:10.1186/s40164-023-00417-y)

**Additional file figures**

**Additional file 1: Figure S1** Correlation between mutation count and tumor size evaluated by CT.

**Additional file 1: Figure S2** Mutation landscape of all enrolled patients.

**Additional file 1: Figure S1** Correlation between mutation count and tumor size evaluated by CT.


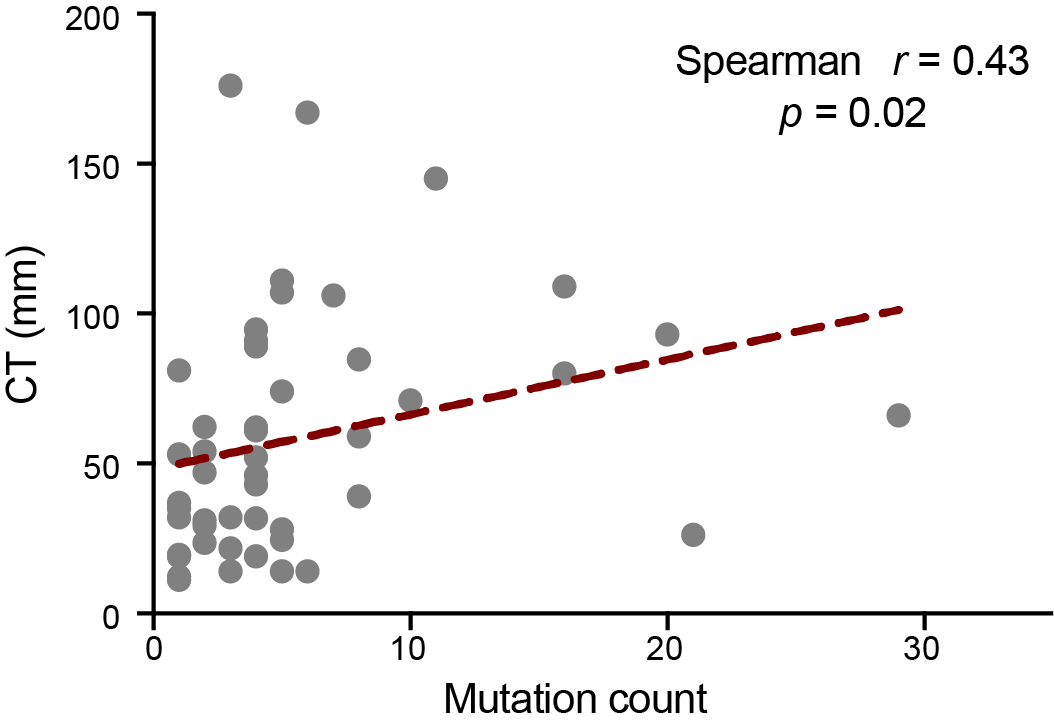


**Additional file 1: Figure S2** Mutation landscape of all enrolled patients. PR, partial response; SD, stable disease; NA, not available.


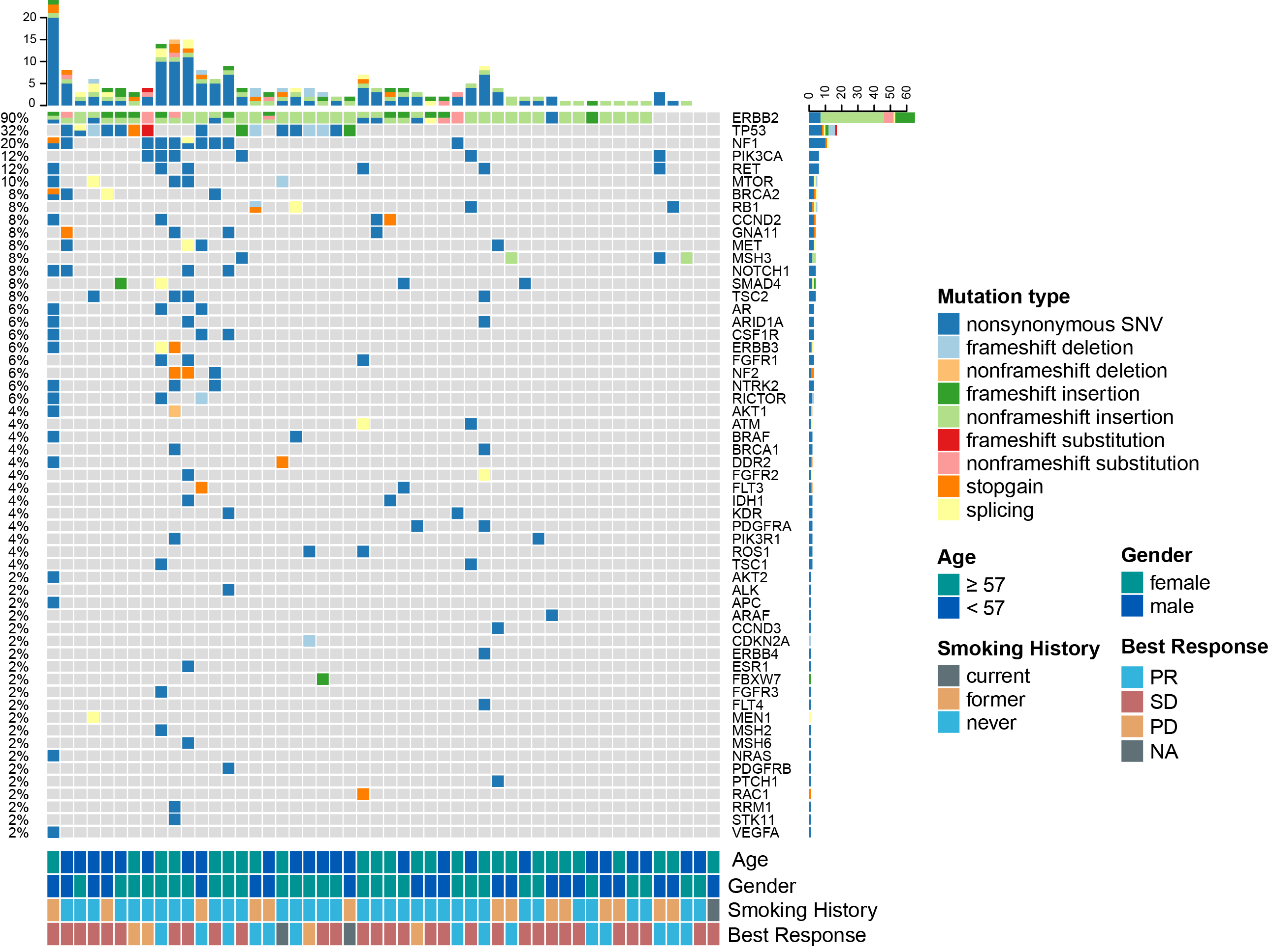

Supplement: Supplementary file 1 — Additional file 1: Figure S1 Correlation between mutation count and tumor size evaluated by CT. Figure S2 Mutation landscape of all enrolled patients. [file 40164_2023_417_MOESM1_ESM.docx]
