# Supplementary material for: Molecular correlation of response to pyrotinib in advanced NSCLC with HER2 mutation: biomarker analysis from two phase II trials
Source: Exp Hematol Oncol. 2023 Jun 9;12:53. doi: 10.1186/s40164-023-00417-y (PMC10251549; doi:10.1186/s40164-023-00417-y)
Supplement: Supplementary file 2 — Additional file 2: Table S1 Comparing demographic data and clinical characteristics between patients with shedding or nonshedding tumor. [file 40164_2023_417_MOESM2_ESM.docx]

**Additional file table**

**Additional file 1: Table S1** Comparing demographic data and clinical characteristics between patients with shedding or nonshedding tumor.

**Additional file 1: Table S1** Comparing demographic data and clinical characteristics between patients with shedding or nonshedding tumor.

| **Characteristic** | **shedding (n=45), No (%)** | **nonshedding (n=5), No (%)** | ***p* value** |
| --- | --- | --- | --- |
| **Median age, years (range)** | 58 (40-72) | 57 (49-63) | 0.582 |
| **Sex** |  |  |  |
| male | 18 (40.0) | 3 (60.0) | 0.638 |
| female | 27 (60.0) | 2 (40.0) |  |
| **Smoking histology** |  |  |  |
| never | 33 (73.3) | 2 (40.0) | 0.074 |
| former | 12 (26.7) | 2 (40.0) |  |
| current | 0 (0.0) | 1 (20.0) |  |
| **ECOG performance status** |  |  |  |
| 0 | 5 (11.1) | 2 (40.0) | 0.138 |
| 1 | 40 (88.9) | 3 (60.0) |  |
| **Clinical stage** |  |  |  |
| IIIB | 2 (4.4) | 0 (0.0) | 1.000 |
| IV | 43 (95.6) | 5 (100.0) |  |
| **No. of metastatic organs** |  |  |  |
| ≤ 2 | 21 (46.7) | 4 (80.0) | 0.349 |
| > 2 | 24 (53.3) | 1 (20.0) |  |
| **Prior chemotherapy** |  |  |  |
| < 2 lines | 24 (53.3) | 4 (80.0) | 0.368 |
| ≥ 2 lines | 21 (46.7) | 1 (20.0) |  |
| **Previous targeted therapy** |  |  |  |
| no | 34 (75.6) | 4 (80.0) | 1.000 |
| yes | 11 (24.4) | 1 (20.0) |  |
| **Previous radiotherapy** |  |  |  |
| no | 31 (68.9) | 4 (80.0) | 1.000 |
| yes | 14 (31.1) | 1 (20.0) |  |
| Abbreviations: ECOG PS, Eastern Corporation Oncology Group. | | | |
